# miR-30e-5p and miR-15a Synergistically Regulate Fatty Acid Metabolism in Goat Mammary Epithelial Cells via *LRP6* and *YAP1*

**DOI:** 10.3390/ijms17111909

**Published:** 2016-11-16

**Authors:** Zhi Chen, Huiling Qiu, Liuan Ma, Jun Luo, Shuang Sun, Kang Kang, Deming Gou, Juan J. Loor

**Affiliations:** 1 Shanxi Key Laboratory of Molecular Biology for Agriculture, College of Animal Science and Technology, Northwest A&F University, Yangling 712100, Shaanxi, China; chenzhijerom@163.com (Z.C.); luckysunshuang@126.com (S.S.); 2 College of Life Sciences, Shenzhen University, Shenzhen 518060, China; 18938944369@163.com (H.Q.); wind2596358@yahoo.com (L.M.); kangkang@szu.edu.cn (K.K.); 3 Mammalian NutriPhysioGenomics, Department of Animal Sciences, Division of Nutritional Sciences, University of Illinois, Urbana, IL 61801, USA; jloor@illinois.edu

**Keywords:** miR-30e-5p, miR-15a, fat metabolism, *LRP6*, *YAP1*

## Abstract

MicroRNA (miRNA) regulates the expression of genes and influences a series of biological processes, including fatty acid metabolism. We screened the expression of miRNA in goat mammary glands during peak-lactation and non-lactating (“dry”) periods, and performed an in vitro study with goat mammary epithelial cells (GMEC) prior to sequencing analysis. Results illustrated that miR-30e-5p and miR-15a were highly expressed. Utilizing a luciferase reporter assay and Western blot, low-density lipoprotein receptor-related protein 6 (*LRP6*) and *Yes associated protein 1* (*YAP1*) genes were demonstrated to be a target of miR-30e-5p and miR-15a in GMEC. Moreover, we demonstrated that the overexpression of miR-30e-5p and miR-15a in GMEC promoted fat metabolism while their knockdown impaired fat metabolism. These findings extend the discovery of a key role of miR-30e-5p and miR-15a in mediating adipocyte differentiation by suggesting a role in promoting milk fat synthesis. In conclusion, our findings indicate that miR-30e-5p, together with miR-15a, represses expression of *LRP6* and promotes fat metabolism in GMEC. The data expanded our knowledge on the function of miRNAs in milk fat metabolism and synthesis in ruminant mammary cells.

## 1. Introduction

Goat milk contains high concentrations short-chain fatty acids and unsaturated fatty acids, which can elicit positive effects on human health [1,2,3]. Thus, investigating the control of goat milk fat metabolism is of great significance [4,5]. Although the focus in the last few decades has been on the analysis of a single gene or function, more comprehensive studies of molecular regulation of milk fat metabolism are needed [6,7,8]. MicroRNA are members of the non-coding RNA family, and are composed of 21–23 nucleotides [9,10,11]. They can negatively regulate target genes via mRNA splicing and inhibiting protein translation [12,13,14,15]. Currently, hundreds of miRNA have been uncovered in non-ruminants, and bioinformatics predictions indicate that they could regulate the expression of approximately thirty percent of the genes in the genome [16,17,18,19]. Previous research indicates that over 60% of protein-coding genes have more than one conserved site for miRNA [1,20]. Various reports illustrated that miRNA were expressed in a spatio-temporal specific manner, and influence a variety of procedures, such as immune regulation, fat metabolism, cell apoptosis, cell proliferation, and cell differentiation, among others. Despite the vast amount of work on miRNA in non-ruminant species [21], there are few studies focused on the function and mechanisms whereby miRNA synergistically regulates the process of milk fat metabolism [22,23,24]. In addition, mechanisms of miRNA working in concert with other miRNA are likely to be uncovered.

By working in concert with transcription factors, and promoting the expression of certain genes, Yes-associated protein (YAP) and its downstream proteins in the Hippo signaling pathway, play a crucial role in promoting cell growth and inhibiting apoptosis. The protein encoded by *LRP6* is a component of the cell-surface receptors for Wnt proteins, and Wnt is known to promote recruitment of Axin via *LRP6* all of which leads to inhibiting degradation of *β-catenin* [25]. Increasing evidence demonstrates that the Hippo/YAP and Wnt/-catenin pathways (crosstalk) control the growth and development of cells, tissues, and organs through various types of interactions [26,27]. Studying the mechanism of the two pathways and the interaction with miRNA will provide a new strategy for regulating milk fat metabolism. Our results revealed that miR-30e-5p and miR-15a are highly-expressed not only in the mammary gland of goats, but also in goat mammary epithelial cells (GMEC) cultured with prolactin. Furthermore, miR-30e-5p cooperates with miR-15a promoting fat metabolism by repressing the expression of *YAP1* in GMEC. Our research illustrates that miR-30e-5p and miR-15a play a significant role in the accumulation of milk fat in ruminants.

## 2. Results

### 2.1. Screening of miRNA at Peak-Lactation and the Non-Lactating Period and Prolactin Effects on miRNA in GMEC

**Figure 1:**
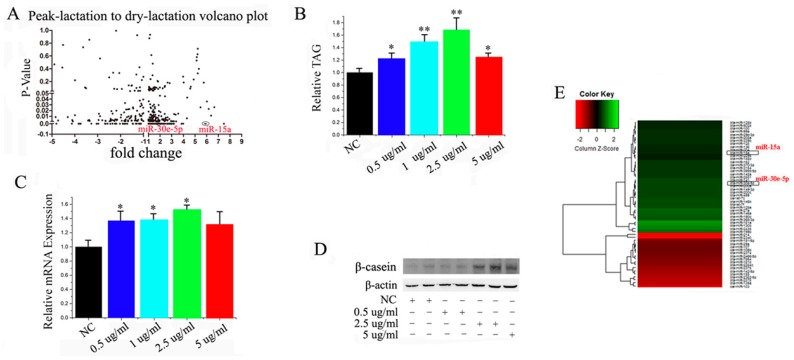
(**A**) Screening of microRNA (miRNA) expressed at peak-lactation and non-lactating stages. Three mammary samples from goats at each stage of lactation were used; (**B**) Triglyceride levels in cells treated with increasing concentrations of prolactin; triglyceride levels were compared with controls (*n* = 6), * *p* < 0.05; ** *p* < 0.01; (**C**) Goat mammary epithelial cells (GMEC) were cultured with prolactin for 48 h, and the expression of *β-casein* was quantified by RT-qPCR (*n* = 6), * *p* < 0.05. All experiments were duplicated and repeated three times. Values are presented as means ± standard errors; (**D**) Western blot analyses of the expression of *β-casein* in the prolactin treatment experiments. The effect of prolactin on *β-casein* protein expression was evaluated by Western blot in GMEC; (**E**) Screening for miRNAs involves in the 0 µg/mL prolactin and 2.5 µg/mL prolactin, respectively. The expression of 18s rRNA is used as a normalization control.

Some reports have showed that miRNA participates in the regulation of milk fat metabolism [28,29,30]. We profiled the differentially-expressed miRNA between peak-lactation and non-lactating periods in goat mammary glands to investigate the connection between miRNA regulation and this physiological process in a more comprehensive way. We applied the S-Poly (T) real method, including 267 Capra hircus primary miRNA and 793 Bos taurus primary miRNA from miRBase. We have underscored the overlapping data and more details can be found in Supplementary Data 1. Firstly, all of the miRNA with four-fold change and *p* < 0.05 were chosen as candidate miRNA. (Figure 1A and Supplementary Data 1). Overall, we identified 55 differentially-expressed miRNA between peak and non-lactating mammary tissue, with 31 up-regulated and 24 down-regulated. To better understand the link between these miRNA and mammary cell growth and milk fat metabolism, we screened miRNA in GMEC cultured with increasing concentrations of prolactin (an essential lactogenic hormone).

In order to detect the expression of miRNAs in GMECs, we used optimal concentration of prolactin. We found that 2.5 μg/mL was the most significant one (Figure 1B–D), so 2.5 μg/mL was our experimental concentration. Then we took a second of screening with the same criterion (Figure 1E, Supplementary Data 2). The analysis indicated that miR-30e-5p and miR-15a were highly expressed (Figure 1A,E).

**Figure 2:**
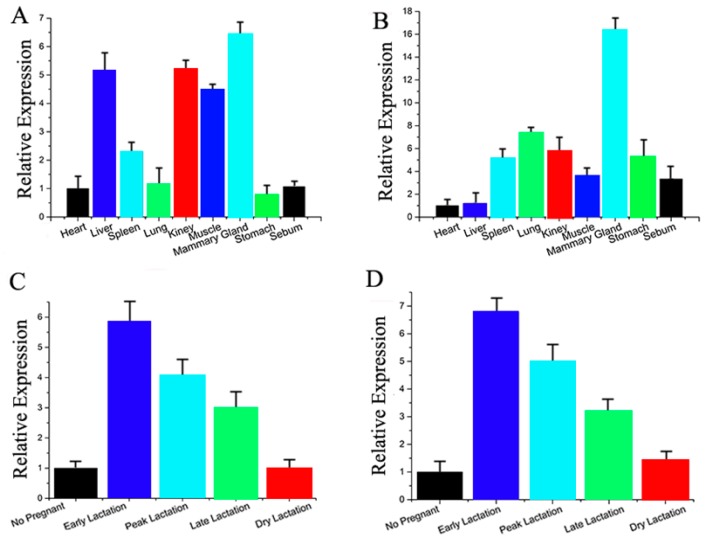
(**A**) miR-30e-5p expression in various tissues of dairy goats; (**B**) miR-15a expression in various tissues of dairy goats; (**C**) miR-30e-5p expression in various tissues of dairy goats; (**D**) miR-15a expression in various tissues of dairy goats. All experiments were duplicated and repeated three times. Values are presented as means ± standard errors

We also performed bioinformatics analysis to screen the differentially-expressed miRNA. Based on the 3′-UTR complementary prediction with Target Scan 6.2 (Whitehead Institute for Biomedical Research, Boston, MA, USA) and miRNA functional analysis by DAVID [31,32], it was evident that many genes are potential targets of the highly-expressed miRNA. Previous studies have revealed that miR-30e-5p and miR-15a are not only associated with milk fat metabolism (Figures S1 and S2; Tables S1 and S2), but their potential target genes appear to have overlapping functions. Therefore, we selected miR-30e-5p and miR-15a for further detailed studies. Next, we measured the expression level of miR-30e-5p and miR-15a in different tissues of dairy goats (Figure 2A,B), and observed that miR-30e-5p and miR-15a were primarily expressed in mammary tissue. Furthermore, we investigated the miRNA expression in mammary glands at different stages of lactation (Figure 2C,D). miR-30e-5p and miR-15a were up-regulated at early lactation and down-regulated thereafter. These results provided strong evidence that miR-30e-5p and miR-15a may play an important role in lactation.

### 2.2. miR-30e-5p, miR-15 Regulate TAG via LRP6 and YAP1

**Figure 3:**
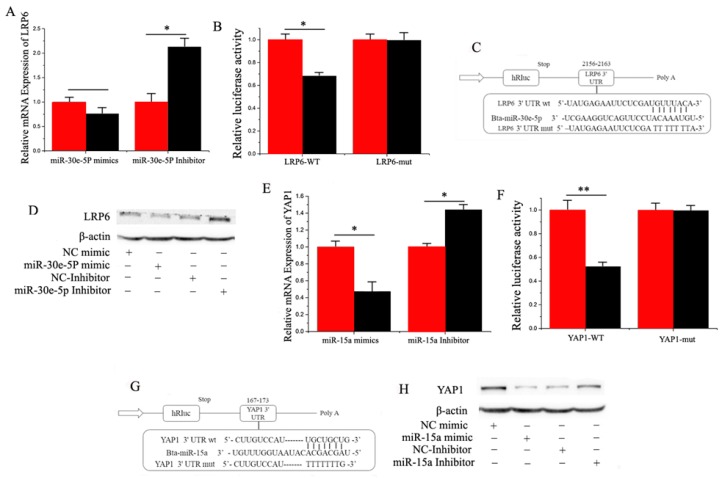
(**A**) RT-qPCR quantification of the *LRP6* expression (*n* = 6). Red bars represent the negative control; black bars represent miR-30e-5p mimic or inhibitor; (**B**,**C**) Target site of miR-30e-5p in *LRP6*3′-UTR and the construction of the luciferase (Luc) expression vector fused with the *LRP6*3′-UTR. WT represents the Luc reporter vector with the WT *LRP6* 3′-UTR (2155–2163); MU represents the Luc reporter vector with the mutation at the miR-30e-5p site in *LRP6* 3′-UTR; (**D**) The effect of miR-30e-5p mimicking and inhibiting *LRP6* protein expression was evaluated by Western blot analysis in GMEC; (**E**) The *YAP1* expression level was quantified by RT-qPCR (*n* = 6). Red bars represent the negative control; black bars represent miR-15a mimic or inhibitor; (**F**,**G**) Target site of miR-15a in the *YAP1*3′-UTR and the construction of the luciferase (Luc) expression vector fused with the *YAP1*3′-UTR. WT represents the Luc reporter vector with the WT *YAP1*3′-UTR (167–173); MU represents the Luc reporter vector with the mutation at the miR-15a site in *YAP1*3′-UTR; (**H**) The effect of miR-15a mimicking and inhibiting *YAP1* protein expression was evaluated by Western blot analysis in GMEC. All experiments were duplicated and repeated three times. Values are presented as means ± standard errors, * *p* < 0.05; ** *p* < 0.01.

Based on target prediction analysis (Target Scan 6.2) and miRNA functional analysis (DAVID), it was evident that many genes are potential targets of miR-30e-5p, including *LRP6*, which encodes a transmembrane cell surface protein involved in receptor-mediated endocytosis [29], and a Wnt co-receptor in the canonical signaling pathway [30]. We chose *LRP6* for functional validation because it is known to be a crucial regulator of multiple metabolic processes, including genetic variations in *LRP6* being associated with high serum LDL (low density lipoprotein) cholesterol levels in humans. Furthermore, Figure 3C illustrates the potential binding sites of miR-30e-5p in the 3′-UTR. We observed that overexpression and inhibition of miR-30e-5p down-regulated and up-regulated the mRNA level of *LRP6*, respectively (Figure 3A).

To verify that miR-30e-5p directly targeted this site, we synthesized a 3′-UTR segment of *LRP6*, including the miR-30e-5p target site, and cloned it into the psi-CHECK2 vector to construct a 3′-UTR reporter plasmid. The luciferase reporter assay indicated that over-expression of miR-30e-5p decreased the relative luciferase activity of the reporter with a wild-type 3′-UTR rather than the one with mutations in the seed sequences (Figure 3B,C). Furthermore, the expression level of the *LRP6* protein was consistent with the mRNA expression data after the miR-30e-5p over-expression treatment (Figure 3D). The findings illustrate that miR-30e-5p directly interacts with the target site of the *LRP6* mRNA and negatively regulates its expression, which partly explained the function of miR-30e-5p during lactation.

Our results also revealed that *YAP1* was down-regulated by overexpression of miR-15a, and was up-regulated by inhibition of miR-15a (Figure 3E). Furthermore, as shown in Figure 3G, goat *YAP1* has a potential binding site for miR-15a in the 3′-UTR. To verify that miR-15a directly targeted this site, we synthesized a 3′-UTR segment of *YAP1* including the miR-15a target site, and cloned it into the psi-CHECK2 vector to construct a 3′-UTR reporter plasmid. The luciferase reporter assay indicated that over-expression of miR-15a decreased the relative luciferase activity of the reporter with a wild-type 3′-UTR rather than the one with mutations in the seed sequences (Figure 3F,G). Furthermore, the expression level of *YAP1* protein was consistent with the expression of mRNA data after the miR-15a over-expression treatment (Figure 3H). The findings illustrated that miR-15a directly interacts with the target site of the *YAP1* mRNA and negatively regulates its expression, which partly explained the function of miR-15a during lactation.

### 2.3. miR-30e-5p Is Associated with β-Catenin and Cooperates with miR-15a in Repressing YAP1 in GMEC

**Figure 4:**
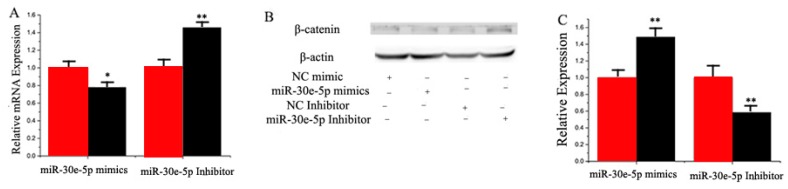

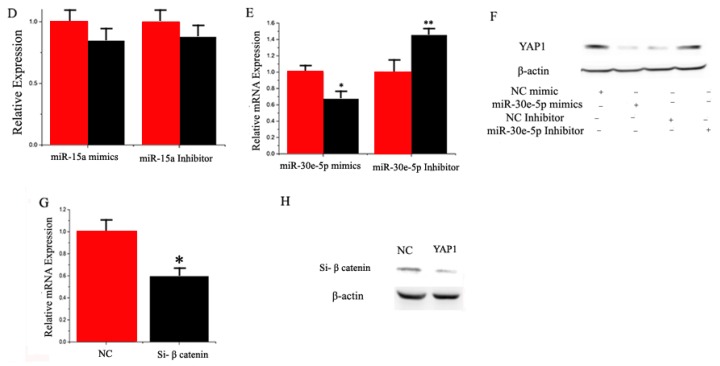
(**A**) RT-qPCR quantification of the *β-catenin* expression level (*n* = 6). Red bars represent the negative control; black bars represent miR-30e-5p mimic or inhibitor; (**B**) The effect of miR-30e-5p mimicking and inhibiting *β-catenin* protein expression was evaluated by Western blot analysis in GMEC; (**C**) GMEC were transfected with miR-30e-5p mimic or inhibitor, and the miR-15a expression level was quantified by RT-qPCR (*n* = 6). Red bars represent the negative control; black bars represent miR-30e-5p mimic or inhibitor; (**D**) GMEC were transfected with miR-15a mimic or inhibitor, and the miR-30e-5p expression level was quantified by RT-qPCR (*n* = 6). Red bars represent the negative control; black bars represent miR-15a mimic or inhibitor; (**E**) the *YAP1* expression level was quantified by RT-qPCR (*n* = 6). Red bars represent the negative control; black bars represent the miR-30e-5p mimic or inhibitor; (**F**) The effect of miR-30e-5p mimicking and inhibiting *YAP1* protein expression was evaluated by Western blot in GMEC; (**G**) GMEC were transfected with Si-NC (60 nM) or SiRNA-*β-catenin* (60 nM) for 48 h, the mRNA expression of *YAP1* was quantified by RT-qPCR (*n* = 6); (**H**) The effect of Si-NC (60 nM) or SiRNA-*β-catenin* (60 nM) for 48 h on *YAP1* protein expression was evaluated by Western blot in GMEC. All experiments were duplicated and repeated three times. Values are presented as means ± standard errors, * *p* < 0.05; ** *p* < 0.01.

Some studies have shown that *LRP6* is an important factor in the Wnt phosphorylation pathway, and *β-catenin* is an important signal mediating Wnt signaling from molecules to the nucleus [25]. Our initial experiment revealed that *LRP6* is a miR-30e-5p’s target gene. To confirm the functional relationship between miR-30e and *β-catenin*, we examined the mRNA level of *β-catenin* using RT-qPCR and Western blot. Figure 4A,B show that there was a significant decrease in *β-catenin* expression in GMEC transfected with a miR-30e-5p mimic in comparison with the control group, indicating that miR-30e-5p did impact the *β-catenin* expression.

Data indicated that miR-30-5p and miR-15a promote fat metabolism. To address the relationship between miR-30-5p and miR-15a we measured the expression of miR-15a in GMEC with miR-30e-5p as being overexpressed or inhibited, respectively. As shown in Figure 4C, compared with the negative control, the expression of miR-15a increased by 1.5 times in the miR-30e-5p mimic transfected cells (*p* < 0.05). On the other hand, the expression of miR-15a decreased significantly when miR-30e-5p was inhibited. However, compared with the negative control, the expression of miR-30e-5p did not change in the miR-30e-5p mimic or inhibited transfected cells (*p* < 0.05) (Figure 4D). The subsequent study analyzed the expression relationship between target genes and miRNA. To resolve this question, we examined the mRNA level of *β-catenin* using RT-qPCR and Western blot. As shown in Figure 4E,F, compared with the negative control, the expression of *YAP1* decreased in miR-30e-5p mimic transfected cells, (*p* < 0.05). On the other hand, the expression of *YAP1* increased significantly when miR-30e-5p was inhibited. Interestingly, the expression of *YAP1* was significantly decreased by Si-*β-catenin*, suggesting a mechanistic relationship between these proteins (Figure 4G,H).

### 2.4. Functional Evaluation of miR-30e-5p, miR-15a, LRP6, and YAP1 in GMEC

The expression of miR-30e-5p was 66 times higher in the miR-30e-5p mimic-transfected GMEC group than the NC (negative control), but expression decreased more than 99% in the miR-30e-5p inhibited group (Figure S3). The expression of miR-15a was 95 times higher in the miR-15a mimic-transfected GMECs than the NC, but expression decreased more than 99% in the miR-15a inhibited group (Figure S4).

**Figure 5:**
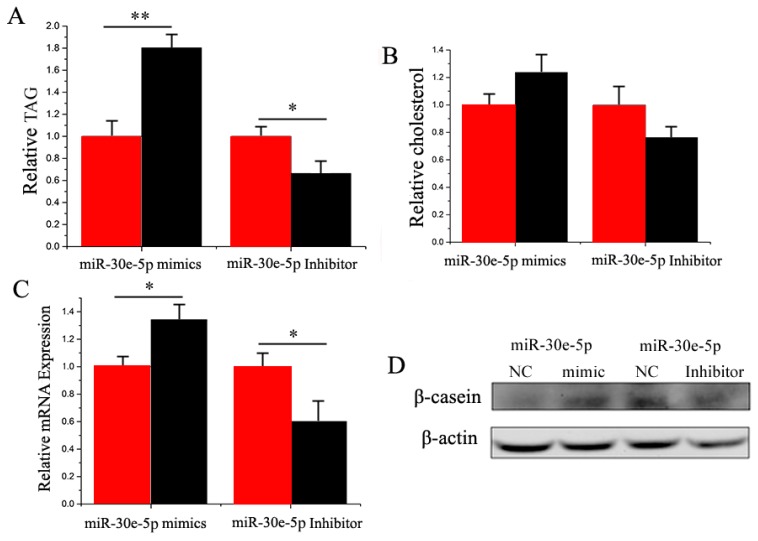
(**A**) Triglyceride levels in cells transfected with miR-30e-5p mimic or inhibitor; Triglyceride levels in transfected cells compared with miR-30e-5p controls (*n* = 6). Red bars represent the negative control; black bars represent miR-30e-5p mimic or inhibitor; (**B**) Cholesterol levels in transfected cells compared with controls (*n* = 6). Red bars represent the negative control; black bars represent miR-30e-5p mimic or inhibitor; (**C**) The expression of *β-casein* was quantified by RT-qPCR (*n* = 6); (**D**): The effect of miR-30e-5p mimic or inhibitor on *β-casein* protein expression was evaluated by Western blot in GMEC. All experiments were duplicated and repeated three times. Values are presented as means ± standard errors, * *p* < 0.05; ** *p* < 0.01.

In GMEC, milk fat exists as lipid droplets composed of TAG (Triglyceride) [32,33]. Compared with the negative control, the TAG content decreased (*p* < 0.05) by 1.8 times in GMEC with the miR-30e-5p mimic (Figure 5A). Similar to TAG, the cholesterol content was increased in miR-30e-5p-transfected cells (Figure 5B). Furthermore, *β-casein* was increased in the miR-30e-5p (Figure 5C,D). Our findings revealed that miR-30e-5p enhanced TAG and cholesterol synthesis in goat mammary cells (Figure 4).

**Figure 6:**
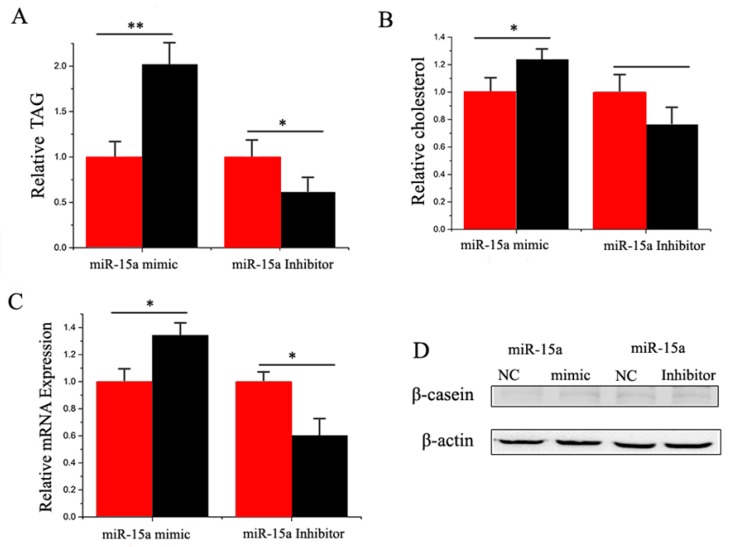
(**A**) Triglyceride levels in cells transfect with miR-15a mimic or inhibitor; triglyceride levels in transfected cells were compared with that of miR-15a controls (*n* = 6). Red bars represent the negative control; black bars represent miR-15a mimic or inhibitor; (**B**) Cholesterol levels in transfected cells were compared with that of control (*n* = 6). Red bars represent the negative control; black bars represent miR-15a mimic or inhibitor; (**C**) The expression of *β-casein* was quantified by RT-qPCR (*n* = 6); (**D**) The effect of miR-15a mimic or inhibitor on *β-casein* protein expression was evaluated by Western blot in GMEC. Total protein was harvested 48 h post-treatment, respectively. All experiments were duplicated and repeated three times. Values are presented as means ± standard errors, * *p* < 0.05; ** *p* < 0.01.

Compared with the negative control, the TAG content increased (*p* < 0.05) by 2.0 times in miR-15a mimic-transfected cells (Figure 6A). On the other hand, the TAG content decreased significantly when miR-15a was inhibited (Figure 6A). Compared with the negative control, the cholesterol content increased (*p* < 0.05) by 1.2 times in miR-15a mimic-transfected cells, but decreased significantly when miR-15a was inhibited (Figure 6B). MiR-15a also enhanced the expression and protein level of milk fat metabolic marker genes (Figure 6C,D). Our findings revealed that miR-15a seems to plays a crucial role in milk TAG synthesis and promotes milk fat metabolism in goat mammary gland (Figure 6).

**Figure 7:**
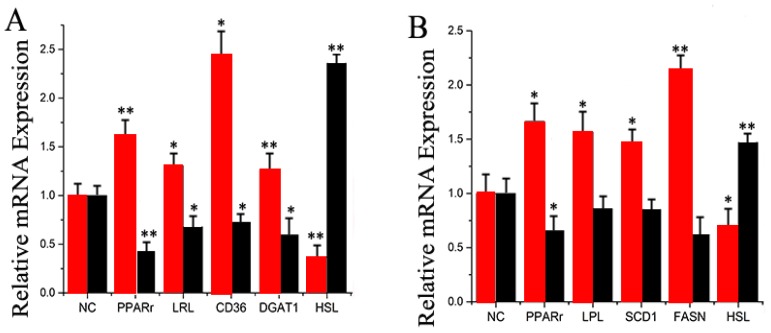
(**A**) Expression of fatty acid metabolism-related genes. The mRNA expression of *PPARγ*, *LPL*, *CD36*, *DGAT1*, and *HSL* was quantified by RT-qPCR (*n* = 6). Red bars represent the miR-30e-5p mimic; black bars represent the miR-30e-5p inhibitor; (**B**) Expression of fatty acid metabolism-related genes. The mRNA expression of *PPARγ*, *LPL*, *SCD1*, *FASN*, and *HSL* was quantified by RT-qPCR (*n* = 6). Red bars represent the miR-15a mimic; black bars represent the miR-15a inhibitor. All experiments were duplicated and repeated three times. Values are presented as means ± standard errors, * *p* < 0.05; ** *p* < 0.01.

Several genes work in a coordinated fashion to control ruminant mammary lipid and protein metabolism [32,33,34]. Ectopic overexpression of miR-30e-5p strongly up-regulated the mRNA expression of *PPARγ*, *LPL*, *DGAT1*, and *CD36* (Figure 7A and Figure S3). In contrast, miR-30e-5p inhibition led to a remarkable down-regulation of *HSL* (Figure 7A and Figure S3). These data indicate that miR-30e-5p plays an important role in the regulation of genes associated with different aspects of fatty acid metabolism.

The results also indicated that ectopic over expression of miR-15a strongly up-regulated the mRNA expression of *PPARγ*, *LPL*, *SCD1*, and *FASN* (Figure 7B and Figure S3). In contrast, cells transfected with the miR-15a inhibitor led to a remarkable down-regulation of a number of genes related to fat metabolism, including *HSL* (Figure 7B and Figure S3). Thus, similar to miR-30e-5p, miR-15a plays an important role in the regulation of genes associated with different aspects of fatty acid metabolism.

**Figure 8:**
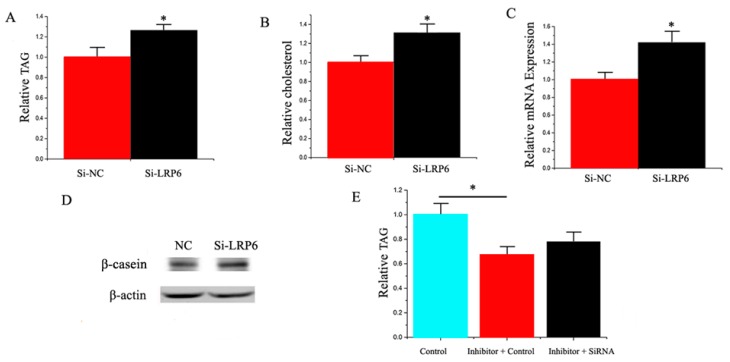
(**A**) Triglyceride levels in cells transfected with Si-NC or SiRNA-*LRP6*; triglyceride levels in transfected cells were compared with Si-NC (*n* = 6). Red bars represent the negative control; black bars represent SiRNA-*LRP6*; (**B**) Cholesterol levels in transfected cells were compared with controls (*n* = 6). Red bars represent the negative control; black bars represent the siRNA-*LRP6*; (**C**) The expression of *β-casein* was quantified by RT-qPCR (*n* = 6); (**D**) The effect of si-NC (60 nM) or siRNA-*LRP6* (60 nM) on *β-casein* protein expression was evaluated by Western blot in GMEC; (**E**) Triglyceride levels in cells transfected with control inhibitor (50 nM) + control siRNA(50 nM), inhibitor 30e-5p (50 nM) + control siRNA (50 nM) and inhibitor 30e-5p (50 nM) + siRNA-*LRP6* (50 nM); triglyceride levels were compared with that of controls (*n* = 6). Values are presented as means ± standard errors, * *p* < 0.05.

Both *LRP6* siRNA and *YAP1* siRNA were used to explore their functional role in GMEC from individual lactating goats. Compared with the negative control, the level of *LRP6* decreased by 75% in GMEC transfected with the *LRP6* siRNA (Figures S5 and S6, Supplementary Data 4). Similarly, compared with the negative control, the *YAP1* level decreased by 72% in the GMEC transfected with *YAP1* siRNA (Figures S5 and S6). As depicted in Figure 8A, compared with the negative control, the TAG content increased (*p* < 0.05) by 1.25 times in GMEC transfected with *LRP6* siRNA. Compared with the negative control, the cholesterol content increased (*p* < 0.05) by 1.2 times in GMEC transfected with *LRP6* siRNA (Figure 8B). In addition, we uncovered that *LRP6* promoted the expression of milk fat metabolic marker genes, including miRNAs and proteins (Figure 8C,D). Our findings revealed that *LRP6* plays a crucial role in milk TAG synthesis and promotes milk fat metabolism in goat mammary cells.

**Figure 9:**
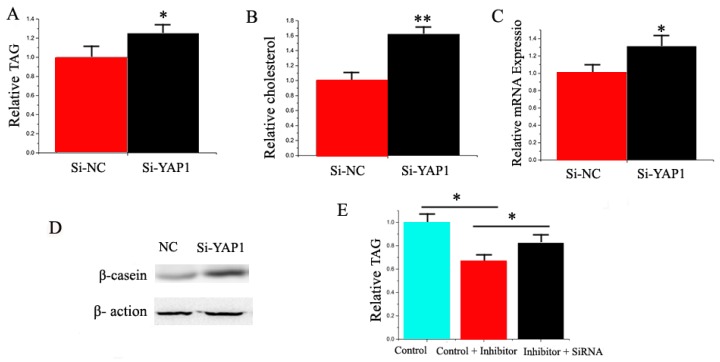
(**A**) Triglyceride levels in transfected cells were compared with that of control (*n* = 6). Red bars represent the negative control; black bars represent miR-15a mimic or inhibitor; (**B**) Cholesterol levels in transfected cells were compared with that of the control (*n* = 6). Red bars represent the negative control; black bars represent siRNA-*YAP1*; (**C**) The expression of *β-casein* was quantified by RT-qPCR (*n* = 6); (**D**) The effect of si-NC (60 nM) or siRNA-*YAP1* (60 nM) on *β-casein* protein expression was evaluated by Western blot in GMEC; (**E**) Triglyceride levels in cells transfected with control inhibitor (50 nM) + control siRNA (50 nM), inhibitor 15a (50 nM) + Control siRNA (50 nM), and inhibitor 15a (50 nM) + siRNA-*YAP1* (50 nM). All experiments were duplicated and repeated three times. Values are presented as means ± standard errors, * *p* < 0.05; ** *p* < 0.01.

Compared with the negative control, the TAG content increased (*p* < 0.05) by 1.35 times in GMEC transfected with *YAP1* siRNA (Figure 9A). Compared with the negative control, the cholesterol content increased (*p* < 0.05) by 1.6 times in GMEC transfected with *YAP1* siRNA (Figure 9B). We also observed that *YAP1* promoted the expression of milk fat metabolic marker genes, including miRNAs and proteins (Figure 9C,D). Our findings revealed that *YAP1* plays a crucial role in milk TAG synthesis and promotes milk TAG synthesis in goat mammary cells.

We applied a “rescue” experiment to demonstrate that miR-30e-5p and miR 15a exert their functions via *LRP6* and *YAP1.* The siRNA-LRP6 rescue increased TAG in GMEC (Figure 8E) in response to the ectopic expression of inhibitor miR-30e-5p. The decrease of TAG was partly alleviated by the siRNA-LRP6 rescue (TAG assay, *p* < 0.05, Figure 7E). Interestingly, the increase of TAG was partly alleviated by the siRNA-*YAP1* rescue (TAG assay, *p* < 0.05, Figure 9E).

## 3. Discussion

### 3.1. miRNA Expression Screening at Peak-Lactation, Non-Lactating Stage, and in Response to Prolactin in GMEC

Previous research illustrated that miRNA plays an important role in mammary development and lactation [35,36,37]. For instance, Avril-Sassen et al. screened and compared the expression of 102 miRNA in different stages of lactation in mice and speculated that some differentially-expressed miRNA were related to fatty acid metabolism [38]. A more recent study using Wendeng goats compared miRNA expression profiles between pregnancy and early lactation and uncovered several differentially-expressed miRNA associated with development and lactation [39]. Lin et al. [40] detected that miR-27 suppresses TAG accumulation in GMEC. However, most of these studies were based on sequencing and array chip technology. The main objective of our study was to better define the relationship of known miRNA in regulating mammary cell fatty acid metabolism. To this end, we screened potential miRNA using differential expression profiling. Overall, we detected 54 differentially-expressed miRNA in peak-lactation and late-lactation stages with 30 up-regulated and 24 down-regulated. In this study, we established the miRNA library including 267 Capra hircus primary miRNAs and 793 Bostaurus primary miRNAs from miRBase, as well as the sequencing results via Solexa sequencing, through which we screened a series of potential miRNA involving in regulation of mammary metabolism. Lin et al. detected that miR-27 suppresses TAG accumulation in GMEC. However, most of these studies were based on sequencing and array chip technology. The main purpose of the present study was to better define the relationship of known miRNA regulating mammary cell fatty acid metabolism. Overall, we detected 54 differentially-expressed miRNA in peak-lactation and non-lactation stages with 30 up-regulated and 24 down-regulated. We established a miRNA library that includes 793 Bos taurus primary miRNAs and 267 Capra hircus primary miRNA from miRBase, with which we screened several potential miRNA involved in regulation of mammary fatty acid metabolism.

To establish which miRNA are associated with the process of growth and development of mammary cells, we cultured GMEC with increasing concentrations of prolactin (a key lactogenic hormone) for subsequent miRNA profiling. We used concentrations of 0 μg/mL, 0.5 μg/mL, 1 μg/mL, 2.5 μg/mL and 5 μg/mL prolactin, and determined that 2.5 μg/mL was the optimal for our specific objective.

### 3.2. miR-30e-5p and miR-15a Target LRP6 and YAP1, Respectively

The protein encoded by *LRP6* is a member of the LDL receptor-related family, a type of transmembrane cell surface protein involved in receptor-mediated endocytosis. *LRP6*, however, is widely known as a Wnt co-receptor in the canonical signaling pathway during embryonic development [30]. Wenzhong Liu suggested that the impairment of Wnt signaling in *LRP6*^+^ mice induced a reduction in body fat mass, diminished hepatic gluconeogenesis, and enhanced BAT and hepatic insulin sensitivity [41]. To confirm that *LRP6* is a direct target of miR-30e-5p, we first cloned the 3′-UTR of *LRP6* for miR-30e-5p into a luciferase reporter plasmid to detect whether miR-30e-5p had a suppressing effect on this gene. Our findings indicated that miR-30e-5p significantly inhibited the luciferase activity, suggesting that miR-30e-5p functions through the 3′-UTR of *LRP6* to inhibit the reporter gene expression. Furthermore, we made a mutation of the potential binding site for miR-30e-5p in the 3′-UTR, and this mutation abrogated the suppressive effect of miR-30e-5p on the 3′-UTR of *LRP6*.

QiuYue Liu reported a time-shifted relationship between the expression *Lats2* and two adipocyte markers C/EBP α and *PPARγ*, and an inverse relationship between *Lats2* and transcriptional co-activator *YAP1*, which positively regulated cell growth and proliferation during differentiation in 3T3-L1 pre-adipocytes [42]. Eunjeong Seo demonstrated a Hippo-independent regulation of *YAP1* by *SOX2* that cooperatively antagonized Wnt/β-catenin signaling and regulated *PPARγ* to determine osteogenic or adipocytic fates [25]. To confirm that *YAP1* is a direct target of miR-15a, we first cloned the 3′-UTR of *YAP1* for miR-15a into a luciferase reporter plasmid to detect whether miR-15a had a suppressing effect on this gene. Our findings indicated that miR-15a significantly inhibited the luciferase activity, suggesting that miR-15a functioned through the 3′-UTR of *YAP1* to inhibit the reporter gene expression. Furthermore, we made a mutation of the potential binding site for miR-15a in the 3′-UTR, and this mutation abrogated the suppressive effect of miR-15a a on the 3′-UTR of *YAP1*.

The rescue experiments dealing with siRNA-*LRP6* and siRNA-*YAP1* partly abolished the decrease of the TAG level induced by inhibitor miR-30e-5p and inhibitor miR-15a. Thus, the rescue experiments illustrated that miR-30e-5p and miR-15a work via *LRP6* and *YAP1*, respectively. These data provided the basis for additional research on *LRP6* and *YAP1* to determine the synergistic mechanism regulating fat oxidation in mammary epithelial cells.

### 3.3. Relationship between miR-30e-5p, β-Catenin, and YAP1

Several reports demonstrated the function of the miR-30 family in adipogenesis and inhibition of osteogenesis. Fang Hu et al. found that miR-30b/c is a key regulator of thermogenesis and uncovered a new mechanism underlying the regulation of brown adipose tissue function and the development of beige fat [43]. Wang showed miR-30e regulated adipocyte by directly targeting the Wnt/LRP6/β-catenin/TCF signaling. [44]. However, all of these studies did not explore the functional and mechanistic role of miR-30e-5p in lactation.

*β-catenin* and *LRP6* play an important role in the Wnt pathway. We verified the *LRP6* is one of miR-30e-5p’s target genes, and RT-qPCR and Western blot data revealed that miR-30e-5p could regulate *β-catenin*. Interestingly, when we knocked out *β-catenin* by siRNA and measured *YAP1* expression, it was surprising to find that *β-catenin* inhibited the expression of *YAP1*. In conclusion, our findings indicated that *β-catenin* could regulate *YAP1* during fat metabolism in GMEC.

### 3.4. miR-30e-5p Cooperates with miR-15a to Repress YAP1

**Figure 10:**
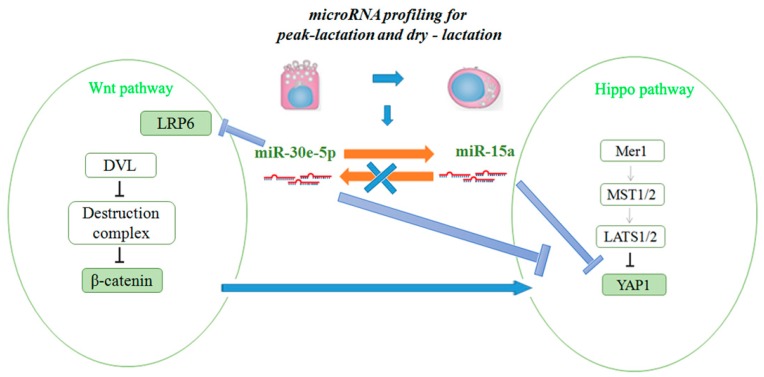
Diagram summarizing our findings: miR-30e-5p and miR-15a synergistically regulate fat metabolism via *LRP6* and *YAP1* in goat mammary epithelial cells.

Although the specific biological roles of some miRNA have been reported, the role that miRNA plays in goat mammary gland epithelial cells is limited. miR-15a/miR-16 control cell cycle in non-small lung cancer cells [45]. Since different tissues expressing miR-15a have different functions [46], we speculated that miR-15a can regulate the role mammary gland by controlling lipid metabolism. When the mimic and inhibitor of miR-15a were cultured in GMEC at the same time, we found that they elicited stronger responses together than they did individually. We also found that miR-30e-5p could regulate miR-15a, but miR-15a could not, in turn, regulate miR-30e-5p. Interestingly, using siRNA, we observed that miR-30e-5p could regulate *YAP1*, which is a target gene of miR-15a, and reduced the expression of *β-catenin* in GMEC (Figure 9). These results suggested that *YAP1* is a factor whose expression is driven by Wnt/*β-catenin* signaling in GMEC. To conclude, we proved that miR-30e-5p could target and regress *LRP6*, which coordinated with miR-15a in regulating *YAP1* and mammary cell fatty acid metabolism (Figure 10).

## 4. Material and Methods

### 4.1. Ethics Statement

The animal use and care protocol was approved by the Animal Use and Care Committee at the Northwest Agricultural and Forestry University, Yang Ling, China.

### 4.2. Animals and RNA Extraction

The elite herd of Xinong Saanen dairy goats used in this research was from the experimental farm of Northwest Agricultural and Forestry University of China. Three healthy goats (three years old) with similar body weight were selected at different stages of lactation: non-pregnant, early-lactation (15 days after parturition), peak-lactation (60 days after parturition), late-lactation (150 days after parturition), and non-lactating (“dry” period). All goats gave birth to kids in the second lactation. Spleen, stomach, heart, liver, sebum, mammary gland tissue, muscle, lung, and kidney were collected after slaughter. Three biological samples per tissue were snap-frozen in liquid nitrogen as soon as possible. Using Trizol reagent (Invitrogen, Carlsbad, CA, USA), total RNA was extracted in accordance with the instructions. The quality and quantity of RNA were detected by a ND-1000 spectrophotometer (NanoDrop, Waltham, MA, USA) and the RNA was stored in −80 °C before experimentation. The samples are a mixture of three goats (at the same period) in non-pregnant, early-lactation (15 days after parturition), peak-lactation (60 days after parturition), late-lactation (150 days after parturition), and non-lactating (“dry” period) stages, respectively.

### 4.3. Cell Culture and Transfection

The GMEC were cultured in DMEM/ F12 medium (Invitrogen, Carlsbad, CA, USA) containing 10% FBS, 10 ng/mL EGF-1 (epidermal growth factor 1, Gibco, Carlsbad, CA, USA), 5 mg/mL insulin, 50 U/mL penicillin/mL streptomycin, and 0.25 mmol/L hydrocortisone in 37 °C in a humidified atmosphere with 5% CO_2_. The GMECs were cultured and fractionated in accordance with a previous study [47]. To induce lactogenesis, GMEC were cultured in a lactogenic medium for 48 h prior to initial experiments [48,49]. On the other hand, GMEC were also processed with 0 μg/mL, 0.5 μg/mL, 1 μg/mL, 2.5 μg/mL, and 5 μg/mL prolactin. Cells were cultured and transfected with either mimic of miR-30e-5p and miR-15a (60 nM) or inhibitor (60 nM) (Invitrogen) using Lipofectamine™ RNAiMAX (Invitrogen) on the basis of the manufacturer’s instructions. Cells were transfected with si-NC (60 nM), siRNA-*LRP6* (60 nM), siRNA-*YAP1*, or siRNA-*β catenin*. Cells were harvested 48 h after transfection.

### 4.4. Assay of Cellular TAG Content 

The GMECs were transfected with either miR-30e-5p and miR-15a mimic or inhibitor. Cells were stained with lysis buffer (1% Triton X-100, pH 7.4, 150 mmol/NaCl, 50 mmol/L Tris-HCL) after 48 h of incubation. TAG was measured using a commercial kit on the basis of manufacturer’s instructions (Loogen, Beijing, China) on an XD 811G Biochemistry Analyzer (Odin Science and Technology Company, Shanghai, China). The values acquired were normalized to the content of total protein with the BCA protein assay kit (Thermo Corp., Waltham, MA, USA, Prod#23227).

### 4.5. Assay of The Cellular Cholesterol Content 

The GMECs were transfected with either miR-30e-5p/miR-15a mimic or inhibitor and siRNA (20 ng). Cells were stained with lysis buffer (1% Triton X-100, pH 7.4, 150 mmol/NaCl, 50 mmol/L Tris-HCL) after 48 h of incubation. Cholesterol was measured using a serum cholesterol kit on the basis of the manufacturer’s instructions (Loogen) on an XD 811G Biochemistry Analyzer (Odin Science and Technology Company, Shanghai, China). The values obtained were normalized to the content of total protein.

### 4.6. RT-qPCR and Western Blot

Both the miRNA detection sensitivity and specificity are improved utilizing the S-Poly (T) assay. In this study, we used the S-Poly (T) real method for the profile expression of 789 miRNA. The expression of 18s rRNA was used as a normalization control. The S1 and S2 were used as specific reverse primers. In brief, reverse transcription was performed as follows: 2.5 μL of 4× reaction buffer, a 10 μL reaction including 0.2 μg total RNA, 1 μL of 0.5 µM RT primer, and 1 μL of poly A/RT enzyme mix. The reaction was performed in 37 °C for 25 min, followed by 42 °C for 25 min, and 72 °C for 10 min. The products of RT were amplified and detected by 20 μL PCR reaction containing 0.3 μL of RT products, a universal Taqman^®^ probe, 0.5 units of Go Taq^®^ Start Polymerase (Promega, Fitchburg, WI, USA), universal reverse primer, 4 μL of 5× qPCR probe Mix, 0.5 µM forward primer and 0.2 mM universal Taqman probe. The PCR reaction was performed at 95 °C for 3 min, followed by 42 cycles at 93 °C for 10 s and 62 °C for 30 s. For the mRNA expression level, 0.3 μg of total RNA were synthesized into cDNA using the PrimeScript^®^ RT Reagent Kit (Perfect Real-Time, Takara, Japan) [49,50,51]. The expression was normalized to UXT. The sequences of primers are listed in Supplementary data 3. All of the real-time reactions, including controls with no templates, were carried out in a Bio-Rad CFX96 real-time PCR detection system (Bio-Rad, Foster City, CA, USA) in triplicate. Relative expression was measured by the 2^−ΔΔ*C*t^ method [40,47,52,53].

As to Western blot analyses, cells were obtained and lysed in RIPA buffer (Solarbio, Beijing, China). Proteins extracted from cells were separated by SDS-PAGE, transferred to nitrocellulose membrane (Millipore, Billerica, MA, USA) and probed with the primary antibodies polyclonal rabbit anti-*LRP6* (Santa Cruz Biotechnology, Santa Cruz, CA, sc-15399, USA), monoclonal rabbit anti-*YAP1* (Cell Signaling Technology Kit#8579), polyclonal rabbit anti-*β-catenin*, rabbit anti-*β-casein* (Biorbyt, orb2053, Wuhan, China), and monoclonal mouse anti-*β-actin* (ProteintechGronup, 66009-1-IG, Wuhan, China), respectively. Polyclonal anti-rabbit HRP-conjugated IgG goat (Tiangen, Beijing, China) was used as a secondary antibody. Signals were detected by the chemiluminescent ECL Western blot system (Pierce, Holmdel, NJ, USA).

### 4.7. Assay of Luciferase Reporter 

To generate reporter constructs for the assay of luciferase, a segment containing a miRNA target site in the 3′-UTR of *LRP6* and YAP1 was inserted into the psi CHECK-2 vector (Promega) between the *NotI* and *Xho* sites immediately downstream of the Renilla luciferase gene. The wild-type segment and mutant-type sequence were established by PCR overlap technology. The sequences of primers were listed in Supplementary Data 5. All constructs were verified using sequencing.

The GMECs were seeded in 384-well plates at a density of 50,000 cells per well one day before transfection. A total of 0.33 g of each reporter construct was transiently transfected using the X-treme GENE HP DNA Transfection Reagent (Roche, Basel, Switzerland) on the basis of the protocol. Cells were then transfected either with mimic or inhibitor using Lipofectamine^TM^ RNAi MAX on the basis of the manufacturer’s protocol after a 6 h recovery period in medium. After 48 h post-transfection, *Firefly* and *Renilla* luciferase activities were measured with the Dual-Glo luciferase assay system on the basis of the manufacturer’s instructions (Promega, Beijing, China).

### 4.8. Statistical Analysis

Analysis of statistics was calculated by the SPSS software package, SPSS 19.0 (Beijing, China). Data are presented as means ± SE (standard error) of three independent experiments. Significant differences were detected when * *p* < 0.05 and ** *p* < 0.01.

## 5. Conclusions

Our results revealed that miR-30e-5p and miR-15 play an important role in fatty acid metabolism in GMEC. In addition, miR-30e-5p not only affected Wnt signaling pathways by regulating *LRP6* and *β-catenin*, but also affects the Hippo signaling pathway by regulating miR-15a and *YAP1* (miR-15 target genes) in GMEC. MiR-30e-5p and miR-15a synergistically regulate fat metabolism via *LRP6* and *YAP1* in goat mammary epithelial cells. In the long-term, these findings might be helpful in developing practical means to improve the quality of ruminant milk.

## Supplementary Materials

Supplementary materials can be found at www.mdpi.com/1422-0067/17/11/1909/s1.

## References

[1] Han J., Lee Y., Yeom K.H., Kim Y.K., Jin H., Kim V.N. (2004). The Drosha-DGCR8 complex in primary microRNA processing. Genes Dev..

[2] Luna P., Bach A., Juarez M., de la Fuente M.A. (2008). Effect of a diet enriched in whole linseed and sunflower oil on goat milk fatty acid composition and conjugated linoleic acid isomer profile. J. Dairy Sci..

[3] Hinrichs J. (2004). Mediterranean milk and milk products. Eur. J. Nutr..

[4] Chilliard Y., Ferlay A., Rouel J., Lamberet G. (2003). A review of nutritional and physiological factors affecting goat milk lipid synthesis and lipolysis. J. Dairy Sci..

[5] Marquart T.J., Allen R.M., Ory D.S., Baldan A. (2010). miR-33 links SREBP-2 induction to repression of sterol transporters. Proc. Natl. Acad. Sci. USA.

[6] Shirasaki T., Honda M., Shimakami T., Horii R., Yamashita T., Sakai Y., Sakai A., Okada H., Watanabe R., Murakami S. (2013). MicroRNA-27a regulates lipid metabolism and inhibits hepatitis C virus replication in human hepatoma cells. J. Virol..

[7] Wilfred B.R., Wang W.X., Nelson P.T. (2007). Energizing miRNA research: A review of the role of miRNAs in lipid metabolism, with a prediction that miR-103/107 regulates human metabolic pathways. Mol. Genet. Metab..

[8] Fabian M.R., Sonenberg N., Filipowicz W. (2010). Regulation of mRNA translation and stability by microRNAs. Annu. Rev. Biochem..

[9] Humphries B., Wang Z., Oom A.L., Fisher T., Tan D., Cui Y., Jiang Y., Yang C. (2014). MicroRNA-200b targets protein kinase Calpha and suppresses triple-negative breast cancer metastasis. Carcinogenesis.

[10] Wang J., Tsouko E., Jonsson P., Bergh J., Hartman J., Aydogdu E., Williams C. (2014). miR-206 inhibits cell migration through direct targeting of the actin-binding protein coronin 1C in triple-negative breast cancer. Mol. Oncol..

[11] Avery-Kiejda K.A., Braye S.G., Mathe A., Forbes J.F., Scott R.J. (2014). Decreased expression of key tumour suppressor microRNAs is associated with lymph node metastases in triple negative breast cancer. BMC Cancer.

[12] Lee Y., Ahn C., Han J., Choi H., Kim J., Yim J., Lee J., Provost P., Radmark O., Kim S. (2003). The nuclear RNase III Drosha initiates microRNA processing. Nature.

[13] Denli A.M., Tops B.B., Plasterk R.H., Ketting R.F., Hannon G.J. (2004). Processing of primary microRNAs by the Microprocessor complex. Nature.

[14] Ying X., Wei K., Lin Z., Cui Y., Ding J., Chen Y., Xu B. (2016). MicroRNA-125b Suppresses Ovarian Cancer Progression via Suppression of the Epithelial-Mesenchymal Transition Pathway by Targeting the SET Protein. Cell. Physiol. Biochem..

[15] Cabello P., Pineda B., Tormo E., Lluch A., Eroles P. (2016). The Antitumor Effect of Metformin Is Mediated by miR-26a in Breast Cancer. Int. J. Mol. Sci..

[16] Sharma S.B., Lin C.C., Farrugia M.K., McLaughlin S.L., Ellis E.J., Brundage K.M., Salkeni M.A., Ruppert J.M. (2014). MicroRNAs 206 and 21 cooperate to promote RAS-extracellular signal-regulated kinase signaling by suppressing the translation of RASA1 and SPRED1. Mol. Cell. Biol..

[17] Filho C.M.C., Calvano-Mendes D.C., Carvalho K.C., Maciel G.A., Ricci M.D., Torres A.P., Filassi J.R., Baracat E.C. (2014). Triple-negative and luminal A breast tumors: Differential expression of miR-18a-5p, miR-17-5p, and miR-20a-5p. Tumour Biol..

[18] Gasparini P., Cascione L., Fassan M., Lovat F., Guler G., Balci S., Irkkan C., Morrison C., Croce C.M., Shapiro C.L. (2014). microRNA expression profiling identifies a four microRNA signature as a novel diagnostic and prognostic biomarker in triple negative breast cancers. Oncotarget.

[19] You L., Pan L., Chen L., Gu W., Chen J. (2016). MiR-27a is Essential for the Shift from Osteogenic Differentiation to Adipogenic Differentiation of Mesenchymal Stem Cells in Postmenopausal Osteoporosis. Cell. Physiol. Biochem..

[20] Gregory R.I., Yan K.P., Amuthan G., Chendrimada T., Doratotaj B., Cooch N., Shiekhattar R. (2004). The Microprocessor complex mediates the genesis of microRNAs. Nature.

[21] Fatima A., Morris D.G. (2013). MicroRNAs in domestic livestock. Physiol. Genom..

[22] Yin H., Pasut A., Soleimani V.D., Bentzinger C.F., Antoun G., Thorn S., Seale P., Fernando P., van Ijcken W., Grosveld F. (2013). MicroRNA-133 controls brown adipose determination in skeletal muscle satellite cells by targeting Prdm16. Cell Metab..

[23] Lee E.K., Lee M.J., Abdelmohsen K., Kim W., Kim M.M., Srikantan S., Martindale J.L., Hutchison E.R., Kim H.H., Marasa B.S. (2011). miR-130 suppresses adipogenesis by inhibiting peroxisome proliferator-activated receptor gamma expression. Mol. Cell. Biol..

[24] Gu Z., Eleswarapu S., Jiang H. (2007). Identification and characterization of microRNAs from the bovine adipose tissue and mammary gland. FEBS Lett..

[25] Seo E., Basu-Roy U., Gunaratne P.H., Coarfa C., Lim D.S., Basilico C., Mansukhani A. (2013). SOX2 regulates YAP1 to maintain stemness and determine cell fate in the osteo-adipo lineage. Cell Rep..

[26] Basu S., Totty N.F., Irwin M.S., Sudol M., Downward J. (2003). Akt phosphorylates the Yes-associated protein, YAP, to induce interaction with 14-3-3 and attenuation of p73-mediated apoptosis. Mol. Cell.

[27] Varelas X., Miller B.W., Sopko R., Song S., Gregorieff A., Fellouse F.A., Sakuma R., Pawson T., Hunziker W., McNeill H. (2010). The Hippo pathway regulates Wnt/beta-catenin signaling. Dev. Cell.

[28] Ye Z.J., Go G.W., Singh R., Liu W., Keramati A.R., Mani A. (2012). LRP6 protein regulates low density lipoprotein (LDL) receptor-mediated LDL uptake. J. Biol. Chem..

[29] Kokubu C., Heinzmann U., Kokubu T., Sakai N., Kubota T., Kawai M., Wahl M.B., Galceran J., Grosschedl R., Ozono K. (2004). Skeletal defects in ringelschwanz mutant mice reveal that Lrp6 is required for proper somitogenesis and osteogenesis. Development.

[30] Hansen H.O., Grunnet I., Knudsen J. (1984). Triacylglycerol synthesis in goat mammary gland, The effect of ATP, Mg2+ and glycerol 3-phosphate on the esterification of fatty acids synthesized de novo. Biochem. J..

[31] DAVID Bioinformatics Resources 6.8. https://david.ncifcrf.gov.

[32] Chen Z., Shi H., Sun S., Xu H., Cao D., Luo J. (2016). MicroRNA-181b suppresses TAG via target IRS2 and regulating multiple genes in the Hippo pathway. Exp. Cell Res..

[33] Bionaz M., Loor J.J. (2008). Gene networks driving bovine milk fat synthesis during the lactation cycle. BMC Genom..

[34] Bionaz M., Loor J.J. (2011). Gene Networks Driving Bovine Mammary Protein Synthesis During the Lactation Cycle. Bioinf. Biol. Insights.

[35] Peng J., Zhao J.S., Shen Y.F., Mao H.G., Xu N.Y. (2015). MicroRNA expression profiling of lactating mammary gland in divergent phenotype swine breeds. Int. J. Mol. Sci..

[36] Bu D.P., Nan X.M., Wang F., Loor J.J., Wang J.Q. (2015). Identification and characterization of microRNA sequences from bovine mammary epithelial cells. J. Dairy Sci..

[37] Zhu J., Sun Y., Luo J., Wu M., Li J., Cao Y. (2015). Specificity protein 1 regulates gene expression related to fatty acid metabolism in goat mammary epithelial cells. Int. J. Mol. Sci..

[38] Avril-Sassen S., Goldstein L.D., Stingl J., Blenkiron C., le Quesne J., Spiteri I., Karagavriilidou K., Watson C.J., Tavare S., Miska E.A. (2009). Characterisation of microRNA expression in post-natal mouse mammary gland development. BMC Genom..

[39] Ji Z., Wang G., Xie Z., Zhang C., Wang J. (2012). Identification and characterization of microRNA in the dairy goat (*Capra hircus*) mammary gland by Solexa deep-sequencing technology. Mol. Biol. Rep..

[40] Lin X.Z., Luo J., Zhang L.P., Wang W., Shi H.B., Zhu J.J. (2013). miR-27a suppresses triglyceride accumulation and affects gene mRNA expression associated with fat metabolism in dairy goat mammary gland epithelial cells. Gene.

[41] Liu W., Singh R., Choi C.S., Lee H.Y., Keramati A.R., Samuel V.T., Lifton R.P., Shulman G.I., Mani A. (2012). Low density lipoprotein (LDL) receptor-related protein 6 (LRP6) regulates body fat and glucose homeostasis by modulating nutrient sensing pathways and mitochondrial energy expenditure. J. Biol. Chem..

[42] Liu Q., Gu X., Zhao Y., Zhang J., Zhao Y., Meng Q., Xu G., Hu X., Li N. (2010). Pig large tumor suppressor 2 (Lats2), a novel gene that may regulate the fat reduction in adipocyte. BMB Rep..

[43] Hu F., Wang M., Xiao T., Yin B., He L., Meng W., Dong M., Liu F. (2015). miR-30 Promotes Thermogenesis and the Development of Beige Fat by Targeting RIP140. Diabetes.

[44] Wang J., Guan X., Guo F., Zhou J., Chang A., Sun B., Cai Y., Ma Z., Dai C., Li X. (2013). miR-30e reciprocally regulates the differentiation of adipocytes and osteoblasts by directly targeting low-density lipoprotein receptor-related protein 6. Cell Death Dis..

[45] Bhattacharya R., Nicoloso M., Arvizo R., Wang E., Cortez A., Rossi S., Calin G.A., Mukherjee P. (2009). miR-15a and miR-16 control Bmi-1 expression in ovarian cancer. Cancer Res..

[46] Bandi N., Zbinden S., Gugger M., Arnold M., Kocher V., Hasan L., Kappeler A., Brunner T., Vassella E. (2009). miR-15a and miR-16 are implicated in cell cycle regulation in a Rb-dependent manner and are frequently deleted or down-regulated in non-small cell lung cancer. Cancer Res..

[47] Shi H., Luo J., Zhu J., Li J., Sun Y., Lin X., Zhang L., Yao D., Shi H. (2013). PPAR gamma Regulates Genes Involved in Triacylglycerol Synthesis and Secretion in Mammary Gland Epithelial Cells of Dairy Goats. PPAR Res..

[48] Peterson D.G., Matitashvili E.A., Bauman D.E. (2004). The Inhibitory Effect of trans-10, cis-12 CLA on Lipid Synthesis in Bovine Mammary Epithelial Cells Involves Reduced Proteolytic Activation of the Transcription Factor SREBP-1. J. Nutr..

[49] Kadegowda A.K.G., Bionaz M., Piperova L.S., Erdman R.A., Loor J.J. (2009). Peroxisome proliferator-activated receptor-γ activation and long-chain fatty acids alter lipogenic gene networks in bovine mammary epithelial cells to various extents. J. Dairy Sci..

[50] Bionaz M., Loor J.J. (2007). Identification of reference genes for quantitative real-time PCR in the bovine mammary gland during the lactation cycle. Physiol. Genom..

[51] Bonnet M., Bernard L., Bes S., Leroux C. (2013). Selection of reference genes for quantitative real-time PCR normalisation in adipose tissue, muscle, liver and mammary gland from ruminants. Animal.

[52] Lin X., Luo J., Zhang L., Zhu J. (2013). MicroRNAs synergistically regulate milk fat synthesis in mammary gland epithelial cells of dairy goats. Gene Exp..

[53] Lin X., Luo J., Zhang L., Wang W., Gou D. (2013). MiR-103 controls milk fat accumulation in goat (Capra hircus) mammary gland during lactation. PLoS ONE.

