# Supplementary material for: miR-30e-5p and miR-15a Synergistically Regulate Fatty Acid Metabolism in Goat Mammary Epithelial Cells via LRP6 and YAP1
Source: Int J Mol Sci. 2016 Nov 16;17(11):1909. doi: 10.3390/ijms17111909 (PMC5133906; doi:10.3390/ijms17111909)
Supplement: Supplementary file 1 [file ijms-17-01909-s001.zip › ijms-150666-Supplementary Materials/ijms-150666-Supplmentary.pdf]

Supplementary Materials: miR-30e-5p and miR-15a Synergistically Regulate Fatty Acid Metabolism in Goat Mammary Epithelial Cells via *LRP6* and *YAP1*

Zhi Chen, Huiling Qiu, Liuan Ma, Jun Luo, Shuang Sun, Kang Kang, Deming Gou and Juan J. Loor

S1: Primers are used for first screening. Screening of miRNAs involves in the peak-lactation and non-lactation.

Capra hircus primary miRNAs, Bos taurus primary miRNAs from miRBase and overlapping data. The samples are mixture of three goats (at the same period) in peak-lactation and non-lactation, respectively. The expression of 18 s rRNA is used as a normalization control.

S2: Primers are used for 2.5 µg/mL prolactin treatment screening.

NC and 2.5 µg/mL experimental concentrations in GMECs are used by S-Poly (T) real-time RT-PCR method to detect. The expression of 18 s rRNA is used as a normalization control.

S3: Primers are used for relative mRNA expression in RT-qPCR assay.

Genes are clustered based on main function relative to milk fat synthesis. GMECs are transfected with mimic or inhibitor for 48 h, the mRNA expression of *PPARγ*, *LPL*, *CD36*, *DGAT1*, *HSL*, *SCD1*, *FASN* is quantified by RT-qPCR. The expression of *UXT* is used as a normalization control.

S4: Primers are used for relative SiRNA-*LRP6*, SiRNA-*YAP1* and SiRNA-*β-casein*.

S5: Primers are used for segment sequence of wild type, and sequence of mutant type is established by PCR technology of overlap.

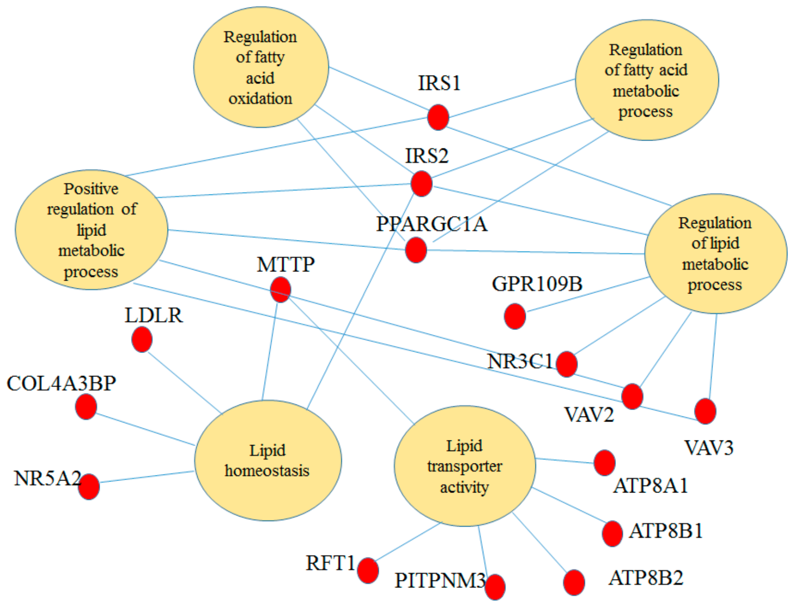

Figure S1. Predicted miR-30e-5p target genes are associated with fat metabolism. All experiments are duplicated and repeated three times.

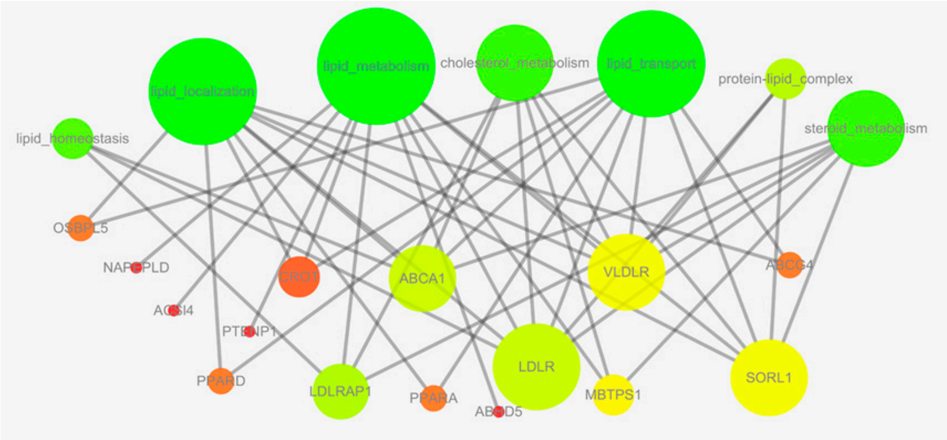

Figure S2. Predicted miR-15a target genes are associated with fat metabolism. All experiments are duplicated and repeated three times.

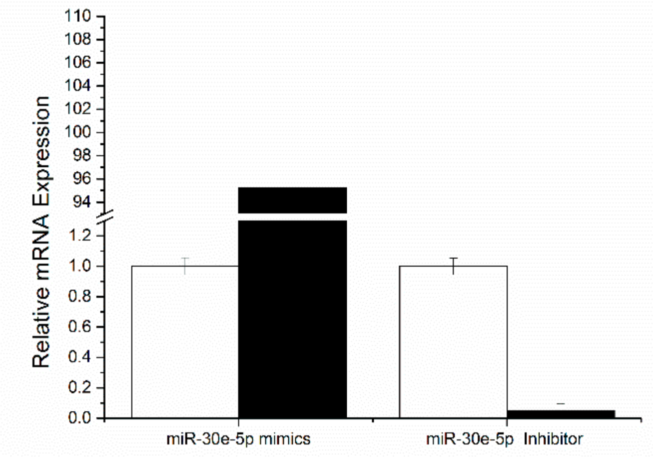

Figure S3. Transfection efficiency of miR-30e-5p mimic and inhibitor. GMECs are transfected with miR-30e-5p mimic or inhibitor for 48 h, and miR-30e-5p expression level is quantified by RT-qPCR (n = 6). White bars represent negative control; black bars represent miR-30e-5p mimic or inhibitor.

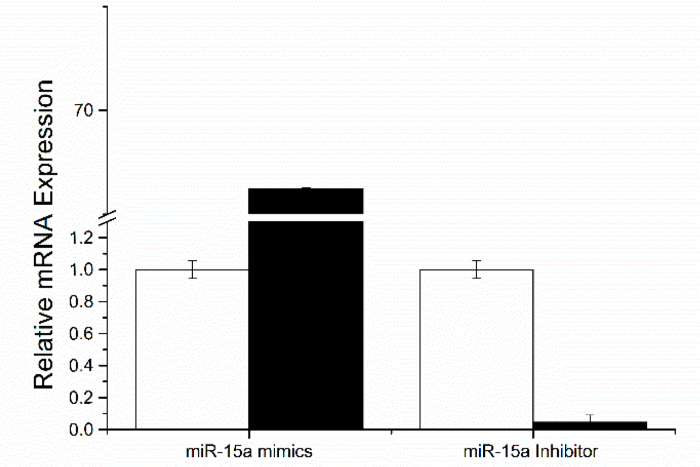

Figure S4. Transfection efficiency of miR-15a mimic and inhibitor. GMECs are transfected with miR-15a mimic or inhibitor for 48 h, and miR-15a expression level is quantified by RT-qPCR (n = 6). White bars represent negative control; black bars represent miR-15a mimic or inhibitor.

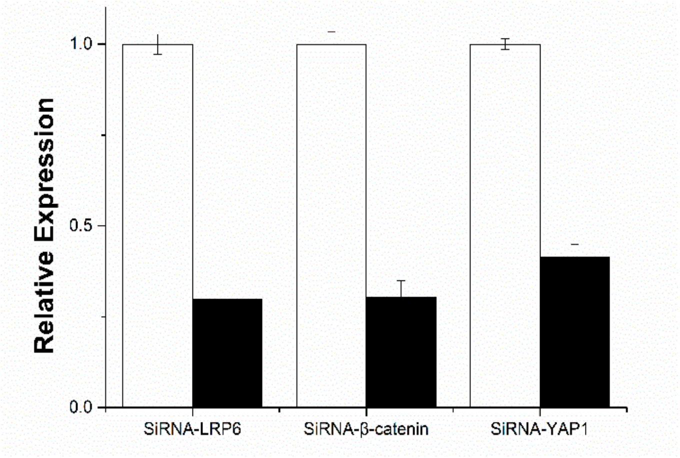

Figure S5. Transfection efficiency of SiRNA. GMECs are transfected with SiRNA-*LRP6*, SiRNA-*YAP1* and SiRNA-*β-casein* for 48 h, and Si-NC (60 nM), SiRNA-*LRP6* (60 nM), SiRNA-*YAP1* (60 nM) and SiRNA-*β-casein* (60 nM) expression levels are quantified by RT-qPCR (n = 6). White bars represent negative control; black bars represent SiRNA.

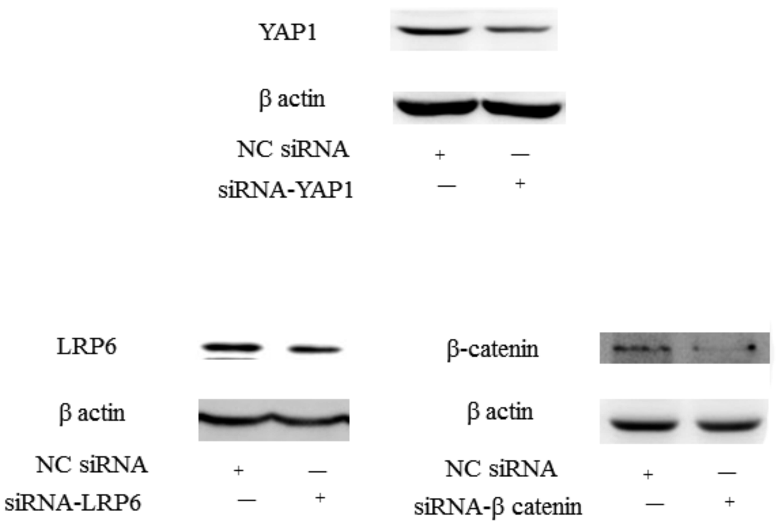

Figure S6. Transfection efficiency of SiRNA. Western blot analyses the expression of *β-casein* in the Si-NC (60 nM), SiRNA-*LRP6* (60 nM), SiRNA-*YAP1* (60 nM) and SiRNA-*β-casein* (60 nM) treatment experiments. The effect of Si-NC (60 nM) or Si-NC (60 nM), SiRNA-*LRP6* (60 nM), SiRNA-*YAP1* (60 nM) and SiRNA-*β-casein* (60 nM) for 48 h on *β-casein* protein expression is evaluated by western blot analysis in GMECs. Total protein is harvested 48 h post-treatment, respectively.

Table S1. Predicted miR-30e-5p target genes are associated with fat metabolism. (The *p*-value is calculated using Fisher's exact test.)

DO Term	Number of Target Genes	*p*-Value
Regulation of fatty acid oxidation	3	5.6×10^{-1}
Regulation of fatty acid metabolic process	3	8.4×10^{-1}
Positive regulation of lipid metabolic process	5	3.5×10^{-1}
Regulation of lipid metabolic process	7	7.5×10^{-1}
Lipid homeostasis	5	4.3×10^{-1}
Lipid transporter activity	6	4.1×10^{-1}

Table S2. Predicted miR-15a target genes are associated with fat metabolism. (The *p*-value is calculated using Fisher's exact test.)

DO Term	Number of Target Genes	*p*-Value
Lipid metabolism	11	3.0×10^{-1}
Cholesterol metabolism	6	2.7×10^{-2}
Steroid metabolism	6	1.2×10^{-1}
Protein-lipid complex	3	5.4×10^{-1}
Lipid transport	9	6.3×10^{-1}
Lipid localization	9	7.2×10^{-1}
Lipid homeostasis	3	8.1×10^{-1}
